# Synergistic and Hepatoprotective Effect of Total Glucosides of Paeony on Ankylosing Spondylitis: A Systematic Review and Meta-Analysis

**DOI:** 10.3389/fphar.2019.00231

**Published:** 2019-03-19

**Authors:** Yao Huang, Hui Wang, Zhe Chen, Yu Wang, Kai Qin, Ying Huang, Pan Shen, Xin Ba, Weiji Lin, Shenghao Tu

**Affiliations:** Institute of Integrated Traditional Chinese and Western Medicine, Tongji Hospital, Tongji Medical College, Huazhong University of Science and Technology, Wuhan, China

**Keywords:** total glucosides of paeony, ankylosing spondylitis, complementary medicine, systematic review, meta-analysis

## Abstract

The objective of this systematic review was to conduct a meta-analysis of the efficacy and safety of total glucosides of paeony (TGP) for the treatment of ankylosing spondylitis (AS). TGP is commonly applied as a complementary medicine, especially in combination with disease-modifying antirheumatic drugs (DMARDs) and/or non-steroidal anti-inflammatory drugs (NSAIDs) to treat AS in China. Nevertheless, the efficacy and safety of TGP combination treatment still needs more validation. A systematic literature search was conducted using PubMed, EMBASE, Web of Science, the Cochrane library, ClinicalTrials, the Chinese Biomedical Literature database (CBM), the China National Knowledge Internet (CNKI), the Wan Fang Medical Database and the VIP Database for available randomized controlled trials (RCTs) investigating the efficacy and safety of TGP on AS up to November 2018. Review Manager 5.3 software and Stata 12.0 software were used to analyze all included studies according to the Preferred Reporting Items for Systematic Reviews and Meta-Analyses (PRISMA) Statement protocol. The pooled results of 23 RCTs exhibited better symptoms improvement (SI) (95% CI 1.16 to 1.36), lower erythrocyte sedimentation rate (ESR) (95% CI −5.89 to −1.32), lower levels of C-reactive protein (CRP) (95% CI −5.01 to −1.49), morning stiffness (MS) time (95% CI −3.46 to −1.86), finger to floor distance (FFD) (95% CI −4.80 to −0.86), peripheral joint pain index (PJPI) (95% CI −3.48 to −0.69), and higher level of thoracic expansion (TE) (95% CI 0.18–0.40) in TGP group. While Schober's test (Schober) showed no significant difference between the two groups. Adverse events (AEs) were significantly decreased (95% CI 0.48–0.79) with the usage of TGP. It is worthwhile to apply TGP as an auxiliary medicine on AS for better efficacy and less side effects, especially when considering the impact of traditional treatment on the liver. Still, further clinical trials with larger sample and better methodological quality are warranted to ascertain the potential benefits of TGP on AS.

## Introduction

Ankylosing spondylitis (AS) is a chronic progressive autoimmune disease of still unknown etiology, characterized by sacroiliitis and enthesitis. If not treated in time, spinal rigidity and deformity may occur in the late stage, which has a serious impact on the quality of patients' life (Braun and Sieper, 2007). According to the 2016 Assessment of SpondyloArthritis international Society (ASAS)- European League Against Rheumatism (EULAR) management, non-steroidal anti-inflammatory drugs (NSAIDs) are recommended as a first-line drug for axial spondyloarthritis. Considering toxicity, contraindications and expenses, conventional synthetic disease-modifying antirheumatic drugs (DMARDs) can be applied and sulfasalazine (SSZ) may be applied to patients with peripheral arthritis (van der Heijde et al., 2017). Thalidomide, a glutamic acid derivative, has the effects of relieving pain, immune regulation, being anti-inflammatory and inhibition of angiogenesis, which also exerts efficacy in the treatment of AS (Yang et al., 2010). When developing a management strategy for AS clinically, weighing the benefits and risks is always needed. However, such therapeutic strategies are either costly or are prone to serious adverse events (AEs). NSAIDs have been reported to increase the risks of cardiovascular, gastrointestinal, and renal effects (van der Heijde et al., 2017). DMARDs like SSZ have reduced patients' compliance due to side effects related to gastrointestinal disorders and skin reactions (Chen et al., 2014). Although TNF inhibitors can be prescribed to patients with persistently high disease activity as recommended by 2016 ASAS/EULAR, they may aggravate infection risk or advance heart failure, lupus as well as cancer (Taurog et al., 2016). Moreover, the high expenses make its application incompatible with developing countries. Thalidomide shows good efficacy compared with DSAIDs, nevertheless, the adverse effect of increasing risk for infertility makes it difficult to be applied widely (Yang et al., 2010). Therefore, it is imperative to explore effective and safer pharmacologic strategies for AS, especially complementary and alternative medicine.

Total glucosides of paeony (TGP) is a water/ethanol extract from the roots of a Chinese herb, Paeonia lactiflora Pallas (also named baishao). It is a biologically active compound predominantly comprising five monoterpene glycosides (paeoniflorin, oxypaeoniflorin, paeonin, albinorin, and benzoylpaeoniflorin). Paeoniflorin accounts for more than 90% and is the predominant pharmacological effector. The Chinese herb, baishao, is prescribed for menstrual disorders and painful diseases like cholecystitis (Zhang and Dai, 2012; Parker et al., 2016). In traditional Chinese medicine (TCM), AS belongs to “Bi” where rich experience has been accumulated during long-term practice. The theory of TCM believes that baishao can relieve pain, nourish blood and soften the liver, which is the indication for “Bi.” Modern clinical trials suggest that TGP can be administrated in various autoimmune disorders like rheumatoid arthritis, primary Sjogren's syndrome, oral lichen planus, alopecia areata and AS, and the intake of TGP causes few AEs (Wang et al., 2007; Yang et al., 2012; Zhou et al., 2016; Jin et al., 2017; Luo et al., 2017). Meanwhile, pharmacological evidence suggests anti-inflammatory, immunoregulatory and analgesic effects of TGP (Jia et al., 2014).

Although TGP has been widely used on AS in China, there are several issues regarding the efficacy and safety of long-term intake of TGP. Given the absence of a systematic review and meta-analysis based on evidence, we conducted this study to comprehensively evaluate the efficacy and safety of TGP combined with DMARDs and/or NSAIDs, presumably allowing clinicians to choose an alternative auxiliary medicine in the treatment of AS.

## Methods

We conducted and reported this review according to the Preferred Reporting Items for Systematic Reviews and Meta-Analyses (PRISMA) Statement protocol (Moher et al., 2015).

### Search Strategy for Identification of Studies

We searched 9 databases including PubMed, EMBASE, Web of Science, the Cochrane library, ClinicalTrials, Chinese Biomedical Literature database (CBM), Wan Fang medical database, the China National Knowledge Internet (CNKI) and the VIP Database in order to ascertain the efficacy and safety of TGP for active AS. All databases were searched to identify all relevant randomized controlled trials (RCTs) published until November 2018.

The key words used were “total glucosides of paeony,” “total glucosides of paony,” “ankylosing spondylitis” and “spondyloarthritis”. For Chinese databases the terms “pa fu lin” OR “bai shao zong ^*^” (for total glucosides of paeony), “qiang zhi xing ji zhu yan” OR “ji zhu xing guan jie yan” (for AS) were used.

### Inclusion Criteria

The inclusion criteria were as follows: (1) Studies used TGP to treat AS. (2) All enrolled patients were diagnosed with AS. (3) There were no other treatment differences between the experimental and control groups. (4) The duration of treatment was at least 3 months (m). (5) The data of interest were available. (6) RCTs. (7) Outcomes included at least one of the following: symptoms improvement (SI), erythrocyte sedimentation rate (ESR), C reactive protein (CRP), thoracic expansion (TE), morning stiffness (MS), finger to floor distance (FFD), Schober test (Schober), peripheral joint pain index (PJPI), and AEs.

### Exclusion Criteria

The exclusion criteria were as follows: (1) Patients were not diagnosed with AS. (2) There were additional treatment factors between the experimental and/or the control group. (3) Incomplete or duplicative data or data of interest were not available. (4) Reviews or cross design trials or comments or case reports.

### Selection of Studies

Two examiners (YaH and HW) independently selected the literature meeting the criteria and extracted relevant data. Discrepancies were resolved by consensus with the corresponding author (SHT).

### Data Extraction and Management

The flow diagram of the study selection was generated according to PRISMA. The essential information of publication year, number of subjects, age, sex, disease duration, dosage, concomitant medication, intervention duration, and outcomes were extracted (Table 1). The primary outcomes were SI and AEs. The secondary outcomes were ESR, CRP, TE, MS, FFD, Schober, and PJPI. If the trial consisted of multiple groups, then only the interested groups were extracted for our meta-analysis. If the data were presented in the form of mode and interquartile range, and the number of cases in each group exceeded 100, then we performed data conversion; if not, the data were abandoned based on the Cochrane Handbook for Systematic Reviews of Interventions.

Table 1The information about all included RCTs.

**Table:** 

**Study**	**Numbers (treatment/control)**	**Age, years (treatment/control)**	**Men, *n* (%) (treatment/control)**	**Disease duration, months (treatment/control)**
Chen, 2004	24/27	31.5	40 (78.4)	NA
Deng et al., 2005	40/40	27.53 ± 10.67/27.70 ± 8.04	34 (85)/33 (82.5)	50.93 ± 56.41/56.20 ± 50.38
Jiang et al., 2012	34/34	25.44 ± 5.43/25.00 ± 5.39	32 (94.1)/31 (91.2)	13.80 ± 4.44/13.68 ± 4.20
Mou and Hu, 2017	36/36	19~43/18~40	33 (91.7)/30 (83.3)	38.14 ± 50.29/33.84 ± 47.15
Li et al., 2006	50/30	NA	69 (86.2)	NA
Li et al., 2014	15/15	26.8 ± 7.4/26.5 ± 6.8	14 (93.3)/14 (93.3)	49.20 ± 31.2/56.4 ± 45.6
Liu et al., 2004	49/49	24 ± 12	78 (79.6)	72 ± 48
Ruan and Zheng, 2013	40/40	23 ± 5.3/22 ± 4.9	33 (82.5)/35 (87.5)	15.0 ± 3.6/14.8 ± 3.7
Su, 2012	21/20	19~43/20~45	20 (95.2)/19 (95)	37.71 ± 58.04/31.67 ± 53.28
Wang and Liu, 2008	29/26	28 ± 7/27 ± 7	27 (93.1)/23 (88.5)	78.00 ± 60.00/72.00 ± 60.00
Wang, 2012	28/28	15~38	32 (57.1)	6.00~120.00
Wang and Cui, 2013	30/30	29.1 ± 3.5	51 (85.0)	55.20 ± 30.00
Wu et al., 2002	30/30	NA	NA	NA
Wu et al., 2014	40/40	34.34 ± 8.21	59 (73.8)	26.52 ± 2.76
Xia and Huang, 2010	22/20	22.4	36 (85.7)	7.00~228.00
Xiong and Tang, 2009	29/29	NA	NA	NA
Yue, 2013	38/32	18~60	55 (78.6)	3.96~120.00
Zhang et al., 2007	28/29	12~60	89 (73.6)	3.00~120.00
Zhang, 2015	44/44	60.3 ± 10.2/58.3 ± 13.2	27 (61.4)/28 (63.6)	NA
Zhang et al., 2017	75/75	24.5 ± 7.1/25.1 ± 6.8	61 (81.3)/62 (82.7)	16.2 ± 5.3/16.7 ± 5.8
Zhao et al., 2003	40/38	25 ± 12	68 (87.2)	72.00 ± 48.00
Zheng et al., 2018	25/25	44.01 ± 6.11/43.29 ± 4.98	22 (88)/21 (84)	16.80 ± 8.28/17.64 ± 6.84
Zou, 2012	60/60	29.2 ± 3.4/30.5 ± 3.7	52 (86.7)/50 (83.3)	56.40 ± 31.20/58.80 ± 26.40

**Table:** 

**Dosage of TGP**	**Concomitant medication**	**Intervention time, months**	**outcomes**
0.6 g bid	SSZ (0.75 g tid) + NSAID (NA)	6	SI, ESR, MS, AEs
0.6 g tid	SSZ (0.5 g tid) + MTX (10 mg qw) + NSAID (0.1 g bid)	3	ESR, CRP, TE, MS, Schober, FFD, AEs
0.6 g tid	LEF (10 mg bid)	12	ESR, AEs
0.6 g tid	SSZ (1 g tid) + MTX (10 mg qw) + NSAID (0.2 g qd)	3	ESR, CRP, TE, MS, Schober, AEs
0.6 g bid	SSZ (0.75 g tid) + MTX (10 mg qw) + NSAID (NA)	6	SI
0.9 g tid/0.6 tid	NSAIDs (NA)	6	ESR, CRP, AEs
0.6 g bid	SSZ (1 g bid)	6	SI, ESR, MS, FFD, AEs
0.6 g tid	Thalidomide (100 mg qd) + MTX (7.5 mg qw) + NSAID (0.1 g bid)	6	ESR, CRP, MS, Schober, PAPI, AEs
0.6 g tid	SSZ (1 g tid) + NSAID (25 mg tid)	6	SI, ESR, CRP, TE, MS, Schober, AEs
0.6 g tid	SSZ (1 g bid)	6	ESR, CRP, TE, MS, Schober, AEs
0.6 g bid	SSZ (0.75 g tid) + NSAID (NA)	6	SI, ESR, CRP, MS, AEs
0.6 g tid	SSZ (1 g tid) + NSAID (0.25 g qd)	6	SI
0.6 g tid	MTX (5 mg qw) + NSAID (0.1 g bid)	3	ESR, CRP, MS, Schober, FFD, AEs
0.6 g tid	Thalidomide (150 mg qd)	3	ESR, CRP, MS, AEs
0.6 g tid	SSZ (0.75 g tid) + NSAID (NA)	6	ESR, CRP, TE, MS, Schober, AEs
0.6 g bid	SSZ (1 g bid) + MTX (10 mg qw) + NSAID (0.75 mg bid)	3	ESR, CRP, TE, MS, Schober, AEs
0.6 g tid	SSZ (0.5 g qid)	6	ESR, CRP, TE, MS, Schober, AEs
0.3 g tid	SSZ (0.75 g tid) + MTX (15 mg qw)	12	ESR, CRP, TE, MS, Schober, FFD, PAPI, AEs
0.6 g tid	MTX (10 mg qw) + NSAID (0.1 g bid)	3	SI, FFD, AEs
0.6 g tid	Thalidomide (150 mg qd) + NSAID (0.25 qd)	6	SI, ESR, CRP, MS, Schober, AEs
0.6 g tid	SSZ (0.75 g tid) + NSAID (75 mg bid)	6	SI, AEs
0.6 g tid	SSZ (1 g bid)	6	CRP, AEs
0.6 g tid	SSZ (1 g tid)	6	ESR, CRP, TE, MS, Schober

*Quantitative data are shown as mean ± SD or median (IQR)*.

*TGP, total glucosides of paeony; SSZ, sulfasalazine; NSAID, non-steroidal anti-inflammatory drug; MTX, methotrexate; LEF, leflunomide; SI, symptoms improvement; ESR, erythrocyte sedimentation rate; CRP, C-reactive protein; TE, thoracic expansion; MS, morning stiffness; Schober's test, Schober; FFD, finger to floor distance; PJPI, peripheral joint pain index; AE, adverse event; NA, not available; SD, standard deviation; IQR, interquartile range*.

### Assessment of Quality in Included Studies

The quality of each included study was assessed according to the Cochrane Collaboration's risk of bias tool consisting of random sequence generation, allocation concealment, blinding of participants and personnel, blinding of outcome assessment, incomplete outcome data, selective reporting, and other bias by two independent reviewers (Higgins and Green, 2011).

### Measures of Treatment Effect

For dichotomous variables, results were summarized using a risk ratio (RR) and a 95% confidence interval (CI), while for continuous outcomes, mean difference (MD), and CI were adopted. Meta-analysis was facilitated by Review Manager 5.3 (Cochrane Collaboration, Oxford, UK).

### Assessment of Heterogeneity

Heterogeneity analyses were conducted by Chi2 and I2 tests. The data were analyzed by a fixed effects model when I2 ≤ 50% or Chi2 test *P* < 0.1, and the random effect model was conducted otherwise.

### Subgroup Analysis

Regardless of the heterogeneity, we performed subgroup analysis which covered intervention time, dosage of TGP, and concomitant medicine. When implementing subgroup analysis of intervention time, data from different time points were included. TGP of 0.3 g tid and 0.6 g bid were deemed as low dosage and 0.6 g tid or 0.9 g tid were high dosage correspondingly. In subgroup analysis, if I2 >50% or Chi2 test *P* > 0.1 in any group, a more conservative random effects model was used.

### Sensitivity Analysis

Sensitivity analysis was conducted to explore the influencing factors and whether the results were robust. Stata 12.0 was used to obtain the figure and Review Manager 5.3 software was used to acquire the precise change of I2 by omitting included trials one by one.

### Assessment of Publication Biases

Publication biases were evaluated through Egger's tests using Stata 12.0 software.

## Results

### Characteristics of Search Results

**Figure 1:**
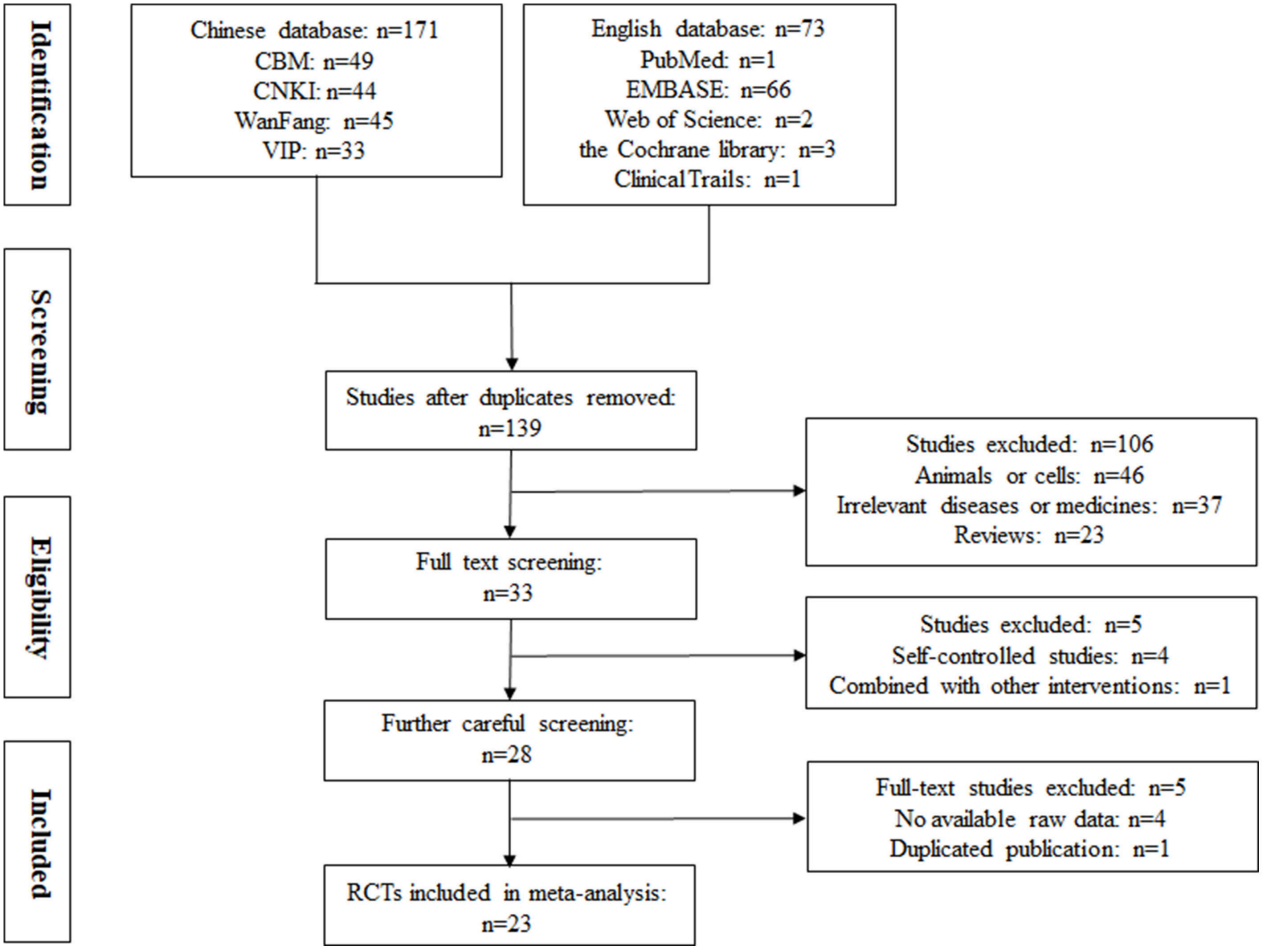
Flow diagram of the study selection process.

As shown in Figure 1, 244 relevant studies were retrieved from nine databases on the basis of the above-mentioned search strategies. After removing duplicates, 139 studies were screened. Then, 106 records were excluded because of animal or cell experiments (*n* = 46), irrelevant diseases or medicines (*n* = 37), or being reviews (*n* = 23). Subsequently, five studies were eliminated owing to self-controlled studies (*n* = 4) and combined with other intervention (*n* = 1). The remaining 28 studies were further searched, and five studies were excluded due to no available raw data (*n* = 4) and duplicated publication (*n* = 1). Finally, 23 RCTs were included for our systematic review and meta-analysis (Wu et al., 2002, 2014; Zhao et al., 2003; Chen, 2004; Liu et al., 2004; Deng et al., 2005; Li et al., 2006, 2014; Zhang et al., 2007, 2017; Wang and Liu, 2008; Xiong and Tang, 2009; Xia and Huang, 2010; Jiang et al., 2012; Su, 2012; Wang, 2012; Zou, 2012; Ruan and Zheng, 2013; Wang and Cui, 2013; Yue, 2013; Zhang, 2015; Mou and Hu, 2017; Zheng et al., 2018).

### Quality of Included Systematic Studies

**Figure 2:**
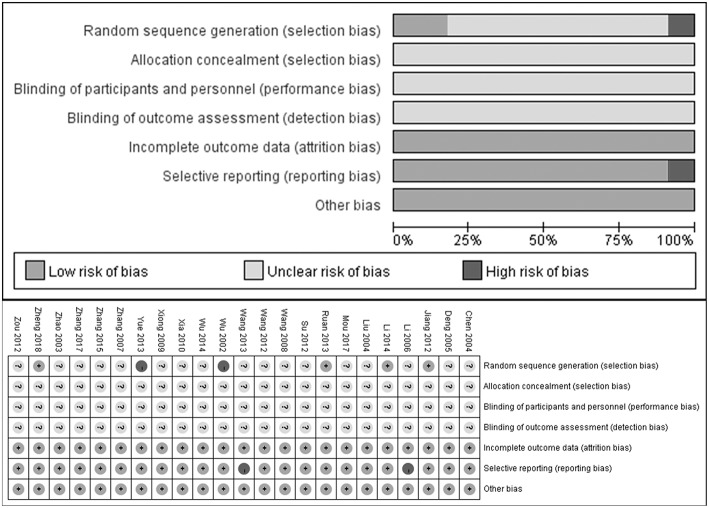
Risk of bias assessment.

As shown in Figure 2, most included studies were of poor methodological quality according to the Cochrane Collaboration's risk of bias tool criteria. 4/23 studies described the generation of random sequence and 2 studies did not mention a random design. Yet none of the included RCTs described the blinded design or the blinding of participants or personnel and outcome assessment. Additionally, 2 of 23 studies did not describe complete outcomes, which was deemed as selective reporting.

### Effects of Interventions

#### The Pooled Efficacy of TGP on AS

**Figure 3:**
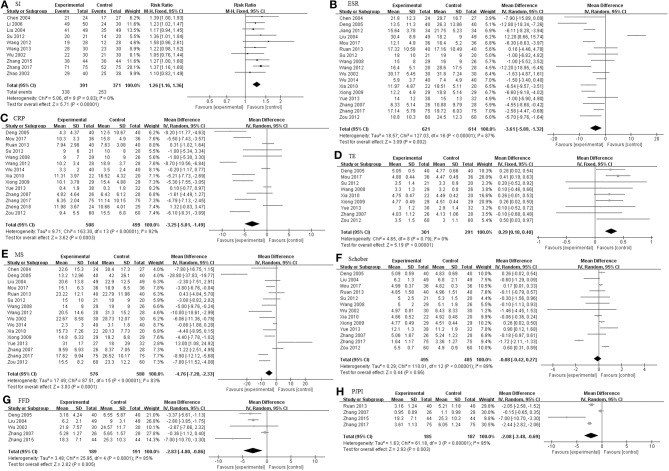
The pooled effects of TGP on AS. Forest plots comparing TGP group and control group. **(A)** SI; **(B)** ESR; **(C)** CRP; **(D)** TE; **(E)** MS; **(F)** Schober; **(G)** FFD; **(H)** PJPI. TGP, total glucosides of peony; AS, ankylosing spondylitis; SI, symptom improvement; ESR, erythrocyte sedimentation rate; CRP, C-reactive protein; TE, thoracic expansion; MS, morning stiffness; Schober, Schober's test; FFD, finger to floor distance; PJPI, peripheral joint pain index.

As illustrated in Figure 3, The pooled results of 23 studies indicated that TGP in combination with other medicines has significant improvements with regard to SI, ESR, CRP, TE, MS, Schober's test, FFD, and PJPI (SI: RR = 1.26, 95% CI 1.16–1.36, *P* < 0.00001; ESR: MD = −3.61 mm/h; 95% CI −5.89 to −1.32; *p* = 0.002; CRP: MD = −3.25 mg/L; 95% CI −5.01 to −1.49; *p* = 0.0003; TE: MD = 0.29; 95% CI 0.18–0.40; *p* < 0.00001; MS: MD = −4.76 min; 95% CI −7.20 to −2.33; *p* = 0.0001; Schober's test: MD = −0.08; 95% CI −0.42–0.27; *p* = 0.66; FFD: MD = −2.83 cm; 95% CI −4.80–−0.86; *p* = 0.005; PJPI: MD = −2.08; 95% CI −3.48 to −0.69; *p* = 0.003) based on a fixed or random effect model.

**Figure 4:**
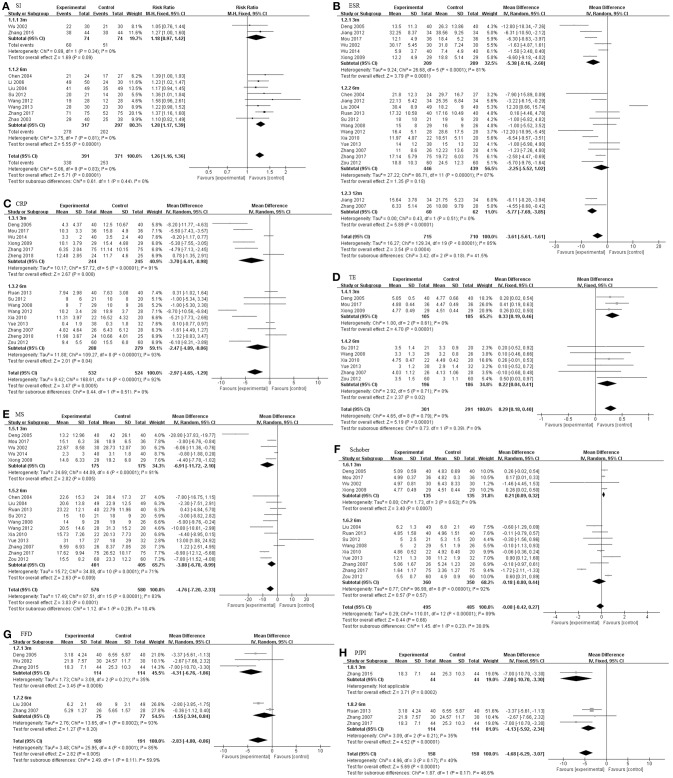
The intervention subgroup of TGP. Forest plots comparing TGP group and control group. **(A)** SI; **(B)** ESR; **(C)** CRP; **(D)** TE; **(E)** MS; **(F)** Schober; **(G)** FFD; **(H)** PJPI. TGP, total glucosides of peony; SI, symptom improvement; ESR, erythrocyte sedimentation rate; CRP, C-reactive protein; TE, thoracic expansion; MS, morning stiffness; Schober, Schober's test; FFD, finger to floor distance; PJPI, peripheral joint pain index.

**Figure 5:**
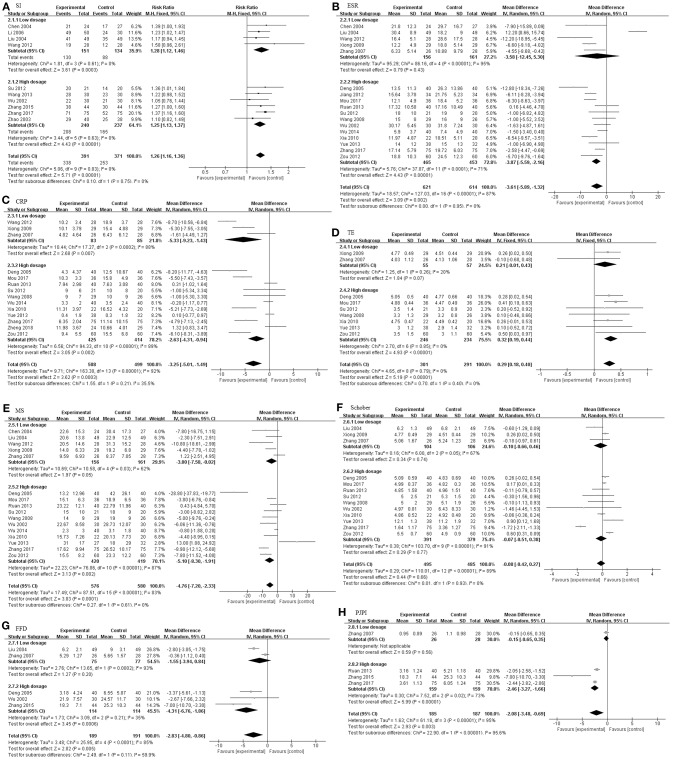
The dosage subgroup of TGP. Forest plots comparing TGP group and control group. **(A)** SI; **(B)** ESR; **(C)** CRP; **(D)** TE; **(E)** MS; **(F)** Schober; **(G)** FFD; **(H)** PJPI. TGP, total glucosides of peony; SI, symptom improvement; ESR, erythrocyte sedimentation rate; CRP, C-reactive protein; TE, thoracic expansion; MS, morning stiffness; Schober, Schober's test; FFD, finger to floor distance; PJPI, peripheral joint pain index.

**Figure 6:**
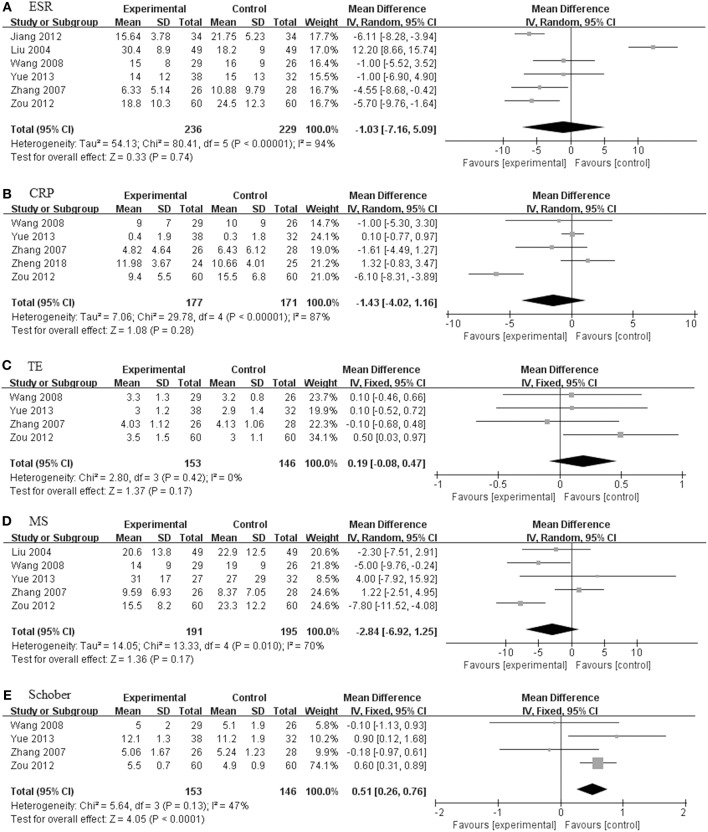
The pooled effects of TGP in combination treatment with DMARDs on AS. Forest plots comparing the TGP group and the control group. **(A)** ESR; **(B)** CRP; **(C)** MS; **(D)** TE; **(E)** Schober. TGP, total glucosides of peony; DMARDs, disease-modifying antirheumatic drugs; AS, ankylosing spondylitis; ESR, erythrocyte sedimentation rate; CRP, C-reactive protein; TE, thoracic expansion; MS, morning stiffness; Schober, Schober's test.

**Figure 7:**
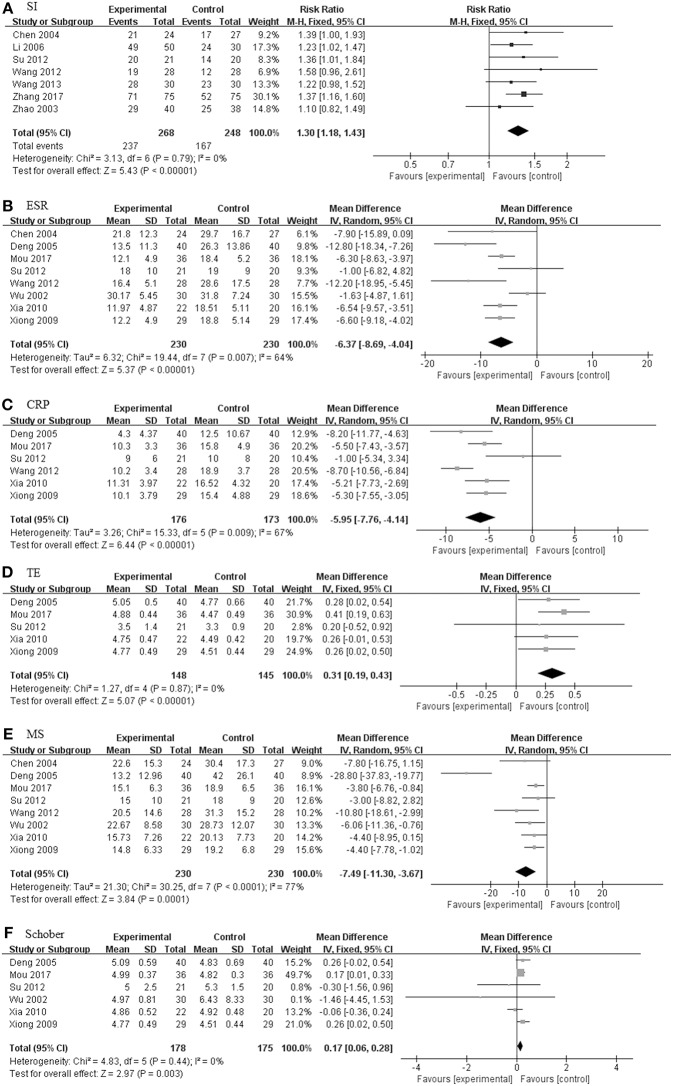
The pooled effects of TGP in combination treatment with DMARDs and NSAIDs on AS. Forest plots comparing the TGP group and the control group. **(A)** SI; **(B)** ESR; **(C)** CRP; **(D)** TE; **(E)** MS; **(F)** Schober. TGP, total glucosides of peony; DMARDs, disease-modifying antirheumatic drugs; NSAIDs, non-steroidal anti-inflammatory drugs; AS, ankylosing spondylitis; SI, symptom improvement; ESR, erythrocyte sedimentation rate; CRP, C-reactive protein; TE, thoracic expansion; MS, morning stiffness; Schober, Schober's test.

We also studied three subgroups according to the study design, including intervention time (Figure 4), dosage of TGP (Figure 5), and the concomitant medicine (Figures 6, 7). In the subgroup of intervention time, superiority occurred in SI and Schober. Compared with 3 m of intervention time (RR = 1.18, 95% CI 0.97–1.42, *P* = 0.09), SI of 6 m group had a slight improvement (RR = 1.28, 95% CI 1.17–1.39, *P* < 0.00001). Similarly, the result of Schober's test was ameliorated along with an increase in time (MD = 0.21; 95% CI 0.09–0.32; *p* = 0.0007 turned to MD = −0.18; 95% CI −0.80 to 0.44; *p* = 0.57). Nevertheless, insignificant impacts were observed on ESR, CRP, TE, MS, and FFD. In the subgroup divided by dosage of TGP, there were important influences on ESR, TE, MS, FFD, and PJPI, while there were few influences on SI, CRP and Schober. There was more favorable evidence for the group of high dosage regarding ESR (MD = −3.87 mm/h; 95% CI −5.59 to −2.16; *p* < 0.00001 vs. MD = −3.58 mm/h; 95% CI −12.45 to −5.30; *p* = 0.43). Increasing the dosage also showed benefit for TE (MD = 0.32; 95% CI 0.19–0.44; *p* < 0.00001 vs. MD = 0.21; 95% CI −0.01 to 0.43; *p* = 0.07). It exhibited slight benefits for MS (MD = −3.80 min; 95% CI −7.58 to −0.02; *p* = 0.05 turned to MD = −5.10 min; 95% CI −8.30 to −1.91; *p* = 0.002) as dosage increased, consistent with FFD, and PJPI (MD = −1.55 cm; 95% CI −3.94 to 0.84; *p* = 0.20 turned to MD = −4.31 cm; 95% CI −6.76 to −1.86; *p* = 0.0006; MD = −0.15; 95% CI −0.65 to 0.35; *p* = 0.56 turned to MD = −2.46; 95% CI −3.27 to −1.66; *p* < 0.00001; respectively). The subgroup of concomitant medicine is displayed below.

#### TGP + DMARDs Compared With DMARDs Alone

There were seven studies (201 in TGP group and 192 in control group) concerning TGP plus DMARDs compared with DMARDs which consisted of SSZ, leflunomide and SSZ in combination with methotrexate (MTX) (Liu et al., 2004; Zhang et al., 2007; Wang and Liu, 2008; Jiang et al., 2012; Zou, 2012; Yue, 2013; Zheng et al., 2018). As illustrated in Figure 6, the pooled results signified that there were no statistically significant differences among ESR, CRP, TE and MS, and values of the difference between the two groups were 1.20 mm/h (95% CI: −7.57 to 9.97), −1.40 mg/L (95% CI: −4.56 to 1.77), 0.28 (95% CI: −0.04 to 0.59), and −2.53 (95% CI: −8.17 to 3.11), respectively. Only the Schober in TGP group was higher than control group (MD = 0.51; 95% CI 0.26–0.76; *p* < 0.0001).

#### TGP + DMARDs + NSAIDs Compared With DMARDs + NSAIDs

Eleven studies including 618 patients (320 in TGP group and 298 in control group) were involved in TGP + DMARDs + NSAIDs compared with DMARDs + NSAIDs, as shown in Figure 7 (Wu et al., 2002; Zhao et al., 2003; Liu et al., 2004; Deng et al., 2005; Li et al., 2006; Xiong and Tang, 2009; Xia and Huang, 2010; Su, 2012; Wang, 2012; Wang and Cui, 2013; Mou and Hu, 2017). With SI, TE and Schober's test under a fixed effects model, and ESR, CRP and MS under a random effects model, pooled results exhibited that TGP had profoundly beneficial effects on all outcomes. The RR of SI between the two groups was 1.30 (95% CI: 1.18–1.43). The MD of ESR was −6.37 mm/h (95% CI: −8.69 to −4.04), consistent with that of CRP (MD: −5.95 mg/L; 95% CI: −7.76 to −4.14). Similarly, the TGP group also showed a positive impact on TE, MS, and Schober's test, values of their MD were 0.31 (95% CI: 0.19 to 0.43), −7.49 (95% CI: −11.30 to −3.67) and 0.17 (95% CI: 0.06 to 0.28).

#### AEs

**Figure 8:**
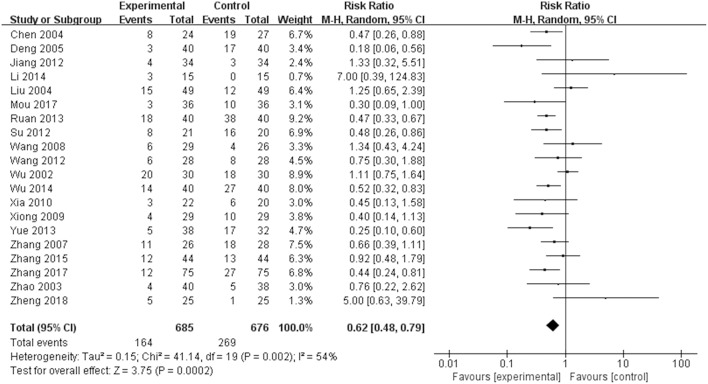
Total AEs of TGP on AS. Forest plots comparing TGP group and control group. TGP, total glucosides of peony; AS, ankylosing spondylitis.

As displayed in Figure 8, 20 studies reported outcomes for AEs. The occurrence in the TGP group was significantly reduced compared with the control group (RR = 0.62, 95% CI 0.48–0.79, *P* = 0.0002). Table 2 lists all AEs that occurred in the included trials. Abnormal liver function was observed in 17 trials (Zhao et al., 2003; Liu et al., 2004; Deng et al., 2005; Zhang et al., 2007, 2017; Wang and Liu, 2008; Xiong and Tang, 2009; Xia and Huang, 2010; Jiang et al., 2012; Su, 2012; Wang, 2012; Ruan and Zheng, 2013; Yue, 2013; Li et al., 2014; Wu et al., 2014; Mou and Hu, 2017; Zheng et al., 2018), and the cases were less frequent in the TGP group compared with the control group. Fourteen trials reported diarrhea (Wu et al., 2002; Zhao et al., 2003; Liu et al., 2004; Wang and Liu, 2008; Xiong and Tang, 2009; Xia and Huang, 2010; Jiang et al., 2012; Su, 2012; Wang, 2012; Ruan and Zheng, 2013; Yue, 2013; Zhang, 2015; Zhang et al., 2017; Zheng et al., 2018), and the occurrence in the TGP group was higher than in the control group. Gastrointestinal disorder was recorded in 12 trials (Wu et al., 2002; Liu et al., 2004; Zhang et al., 2007; Wang and Liu, 2008; Xiong and Tang, 2009; Xia and Huang, 2010; Jiang et al., 2012; Su, 2012; Yue, 2013; Zhang, 2015; Mou and Hu, 2017; Zheng et al., 2018) and the pooled results showed a reduction of gastrointestinal disorder in the TGP group. Leukopenia was less frequent in the TGP group, which was described in six trials (Deng et al., 2005; Xiong and Tang, 2009; Su, 2012; Yue, 2013; Mou and Hu, 2017; Zhang et al., 2017). Four trials (Zhang et al., 2007, 2017; Su, 2012; Yue, 2013) reported lower occurrence of rash in the TGP group. Tingling or numbness in the hands and feet was recorded in three trials (Ruan and Zheng, 2013; Wu et al., 2014; Zhang et al., 2017), and the occurrence was less frequent in the TGP group. Three trials (Wu et al., 2002; Ruan and Zheng, 2013; Zhang, 2015) observed headache, whereas one favored the TGP group and another favored the control group. Insomnia was mentioned in two trials (Wu et al., 2002; Zhang, 2015) and it seemed that TGP could ameliorate insomnia. Two trials (Ruan and Zheng, 2013; Wu et al., 2014) that included the usage of thalidomide described less frequent constipation when thalidomide was given in combination with TGP. A discrepancy was found concerning mucosal ulcer in two trials (Zhang et al., 2007; Ruan and Zheng, 2013). A trial (Wu et al., 2014) also showed occurrences of sleepiness, dizziness and drymouth; cases of sleepiness and drymouth were less in TGP group, but cases of dizziness remained the same between two groups. Another trial (Ruan and Zheng, 2013) indicated that TGP reduced the cases of lower limb edema.

**Table 2:** The AEs about all included trials.

**AEs**	**Experimental**		**Control**	
	**Events**	**Total**	**Events**	**Total**
Patients with AEs	164	685	269	676
Abnormal liver function	21	587	88	575
Diarrhea	52	504	18	490
Gastrointestinal disorder	54	383	81	373
Leukopenia	6	239	14	232
Rash	3	160	6	155
Numbness	4	155	10	155
Headache	7	114	9	114
Insomnia	2	74	3	74
Constipation	1	80	15	80
Mucosal ulcer	5	66	6	68
Sleepiness	10	40	12	40
Dizziness	3	40	3	40
Dry mouth	0	40	1	40
Lower limbs edema	6	40	11	40

*Meta-analysis (fixed effect model or random effect model) was done for these AEs: Patients with AEs: (RR = 0.62, 95% CI 0.48–0.79, P = 0.0002); Abnormal liver function: (RR = 0.26, 95% CI 0.17–0.40, P < 0.00001); Diarrhea: (RR = 2.48, 95% CI 1.53–4.01, P = 0.0002); Gastrointestinal disorder: (RR = 0.67, 95% CI 0.51–0.89, P = 0.005); Leukopenia: (RR = 0.41, 95% CI 0.16–1.05, P = 0.06); Rash: (RR = 0.53, 95% CI 0.15–1.89, P = 0.33). AE, adverse event*.

### Sensitivity Analysis

**Figure 9:**
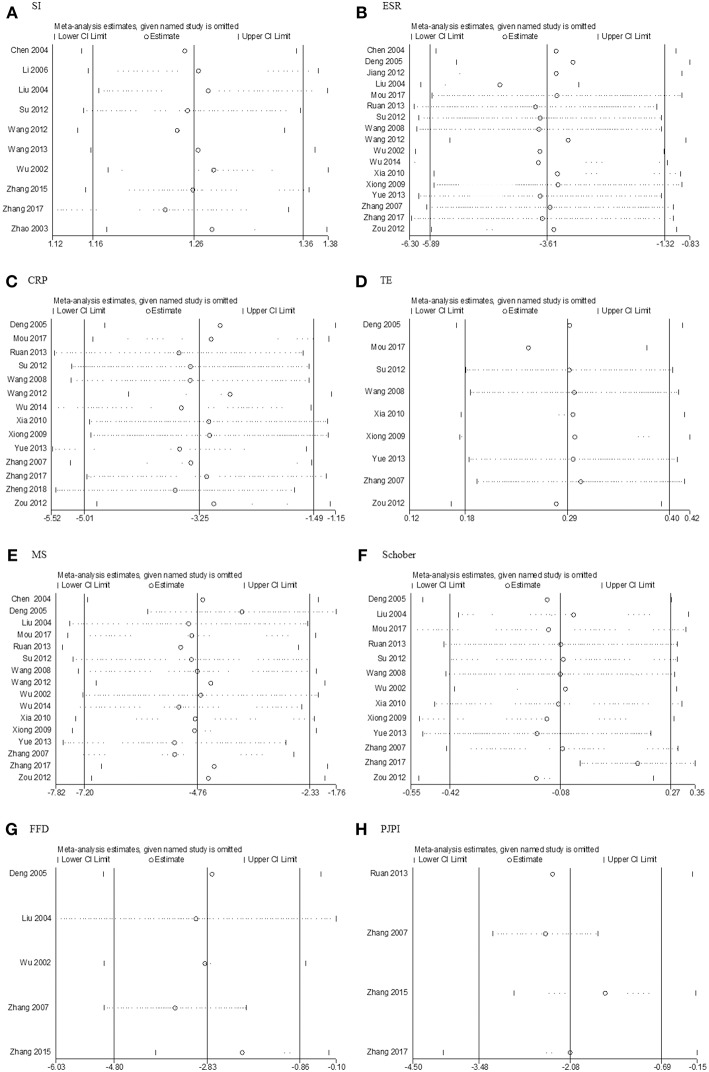
Sensitivity analysis. **(A)** SI; **(B)** ESR; **(C)** CRP; **(D)** TE; **(E)** MS; **(F)** Schober; **(G)** FFD; **(H)** PJPI. SI, symptom improvement; ESR, erythrocyte sedimentation rate; CRP, C-reactive protein; TE, thoracic expansion; MS, morning stiffness; Schober, Schober's test; FFD, finger to floor distance; PJPI, peripheral joint pain index.

There were no significant changes in sensitivity analysis (Figure 9) from SI, ESR, CRP, TE, MS, Schober's test, or PJPI. But for FFD, the heterogeneity was reduced remarkably (I2 reducing from 85–36%) upon the removal of Zhang's 2007 trial (Zhang et al., 2007).

### Publication Bias

**Figure 10:**
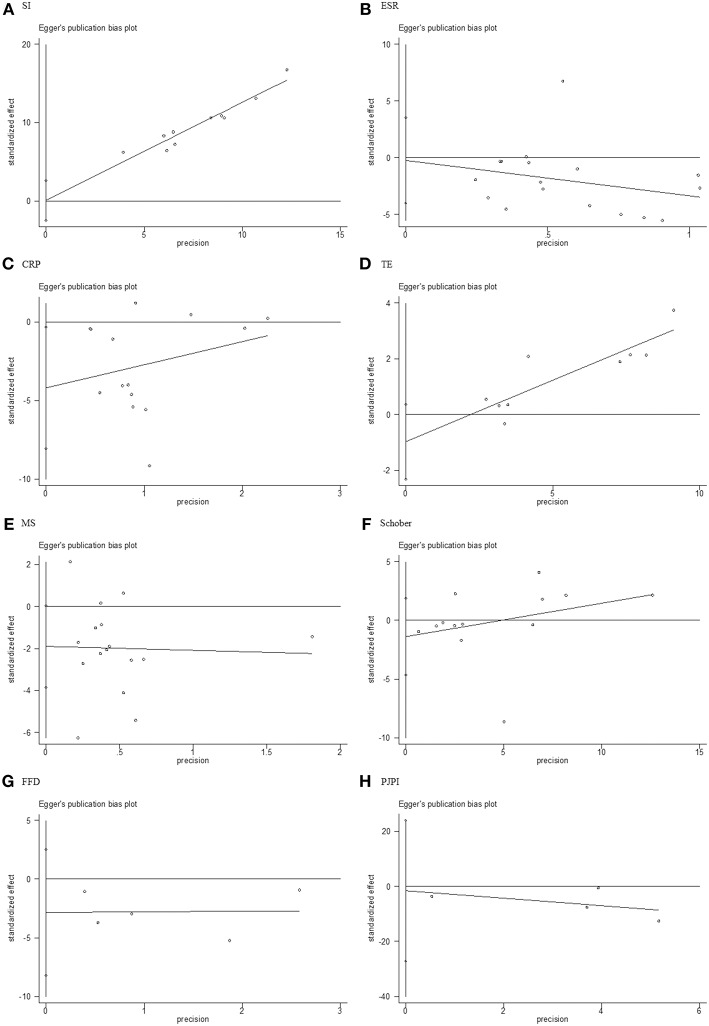
Egger's test for publication bias. **(A)** SI; **(B)** ESR; **(C)** CRP; **(D)** TE; **(E)** MS; **(F)** Schober; **(G)** FFD; **(H)** PJPI. SI, symptom improvement; ESR, erythrocyte sedimentation rate; CRP, C-reactive protein; TE, thoracic expansion; MS, morning stiffness; Schober, Schober's test; FFD, finger to floor distance; PJPI, peripheral joint pain index.

Stata 12.0 software was used to assess publication bias, and Egger's test with *p* > 0.05 was deemed low heterogeneity. The results (Figure 10) of SI (*p* = 0.941), ESR (*p* = 0.893), TE (*p* = 0.13), MS (*p* = 0.055), Schober (*p* = 0.369), FFD (*p* = 0 .190), and PJPI (*p* = 0.812) signified nominal bias aside from CRP (*p* = 0.035).

## Discussion

### Summary of Evidence

Despite significant advances in the treatment of AS, many problems such as high cost and side effects limit their clinical applications. Striking a balance between efficacy and cost is a question that clinicians and patients need to consider carefully. TGP has precise immunomodulatory and anti-inflammatory effects and should be a reasonable complementary choice for the treatment of autoimmune diseases.

This is the first PRISMA-compliant systematic review to assess the efficacy and safety of TGP for patients with AS, which may provide an alternative treatment for AS. The pooled results suggested that TGP adjuvant therapy enhanced SI, reduced the levels of ESR, CRP, MS, FFD, PIPJ, and increased the level of TE, but had little influence on Schober's test, which in general could elucidate that TGP had a significant effect in the treatment of AS. To figure out the potential sources of heterogeneity, subgroups of intervention time, dosage of TGP and concomitant medicine were conducted. As far as intervention time was concerned, improvement was observed in SI and Schober's test. While for the dosage subgroup, outcomes of ESR, TE, MS, FFD, and PJPI were ameliorated as dosage of TGP increased. When stratified with concomitant medicine, TGP+ DMARDs and TGP+ DMARDs+ NSAIDs were carried out. Comparing TGP+ DMARDs with DMARDs alone, ESR, CRP, TE, and MS exhibited no statistical differences, whereas Schober's test had some. Notably, comparing TGP+ DMARDs+ NSAIDs with DMARDs+ NSAIDs, pooled results exhibited that TGP had markedly beneficial effects on all outcomes. Therefore, intervention time, dosage of TGP, and concomitant medicine were factors that were attributed to high heterogeneity. However, efficacy of TGP was not consistent in all outcomes with the increase of intervention time and dosage, and the results were thoroughly different with the usage of NSAIDs. The phenomenon might be explained by several reasons. To begin with, data were not sufficient in each group, making it difficult to synthetize. Additionally, concomitant medicines were various in all groups, which increased the complexity of the subgroups. Thirdly, low quality of included trials may cause subjectivity to a certain degree. Moreover, NSAIDs, as the first-line drug for AS, might exert better efficacy for all outcomes. The fact that only FFD was changed obviously in the sensitivity analysis indicated the robustness and reliability of the results. Taking the possible publication bias into consideration, scrupulous conclusions should be interpreted regarding CRP.

The results of AEs, to our surprise, were decreased considerably in TGP group, among which events of abnormal liver function and gastrointestinal disorder were reduced significantly. And there was no statistically difference regarding rash and leukopenia, only were occurrences of diarrhea increased. A small proportion of patients prescribed with TGP, observed clinically, reported stool changes, such as diarrhea, but generally the situation relieved or disappeared without any treatment or after reducing the dosage. It drew our attention that when TGP was combined with thalidomide, the AE of thalidomide—constipation and the AE of TGP—diarrhea decreased clearly, contributing to an impressive therapeutic regimen on which patients without fertility needs might benefit from attempting.

Increased level of tumor necrosis factor (TNF)-a in the serum, synovium and sacroiliac joints is one of the hallmarks of AS, so the guideline by ASAS-EULAR also recommends TNFi therapy when conventional treatments fail to control inflammation (Ren et al., 2013; van der Heijde et al., 2017). Two trials (Li et al., 2014; Zhang et al., 2017) demonstrated that TGP could effectively reduce expression of TNF-a compared with the control group. The anti-inflammatory property TGP displayed was also substantiated in other clinical and animal experiments, and the underlying mechanisms might be attributed to the reduction of the infiltrated T cells, the regulation of the NF-κB signaling pathway and so on (Chen et al., 2016; Wang et al., 2016).

Cardiovascular disease (CVD) risk in patients with AS is substantially elevated compared with healthy populations, and the intake of NSAIDs is associated with an increased CVD risk (Roubille et al., 2015; Agca et al., 2017). So TGP, a drug also exerting the lipid-regulating effect, may be prescribed as an adjunct combined with NSAIDs (Zheng et al., 2014; Zhang and Fan, 2016). Since the lipid profile has yet to be investigated in clinical trial regarding TGP on AS, we propose more emphasis to be put on this aspect.

## Limitations

Several limitations in the meta-analysis should also be taken into account. First, the limited data of some subgroups might limit the ability to generalize the conclusion. Second, some methodological weaknesses in the primary studies existed, making it prone to produce false positive, or false negative results. Moreover, all included trials were performed in China, which might present a high risk of selection bias. Additionally, high heterogeneity, possibly owing to distinct disease state and concomitant medicine in some results, should be considered seriously when interpreting the results.

## Implications for Practice

Application of TGP has been increasing in the treatment of AS for several years; however, its mechanisms and the efficacy as an adjunct have not been fully recognized among clinicians. Regarding the efficacy-enhancing, hepatoprotective, and potential CVD-reducing effect of TGP, the combination treatment with DMARDs/NSAIDs/thalidomide may provide a quite high performance-to-price ratio option, especially for patients with AS who cannot afford the high expense of biologicals and who are in mild or moderate disease activity. Additionally, it is an indication for patients suffering from constipation. Based on evidence from our meta-analysis, 6 months of course and 0.6 g tid/0.9 g tid of dosage are recommended. If diarrhea occurs in some patients, reduction of dosage or withdrawal can usually be resumed.

## Conclusions

Regarding TGP as an adjunct combined with DMARDs and/or NSAIDs was a more effective and safer therapeutic regiment, TGP is therefore suggested to be considered during the conventional treatment of AS. Potential side effects of the combination treatment also deserve consideration, especially when administrated chronically. Further methodical and rigorous clinical trials and experimental trials elucidating the underlying mechanisms are warranted to verify these conclusions.

## Author Contributions

YaH and HW collected the data and performed the analysis and YaH wrote the article. ZC, YW, and KQ gave advice on the study. YiH, PS, XB, and WL helped to revise the paper. ST solved the discrepancies.

### Conflict of Interest Statement

The authors declare that the research was conducted in the absence of any commercial or financial relationships that could be construed as a potential conflict of interest.
